# Transport Characteristics of Silicon Multi-Quantum-Dot Transistor Analyzed by Means of Experimental Parametrization Based on Single-Hole Tunneling Model

**DOI:** 10.3390/nano13111809

**Published:** 2023-06-05

**Authors:** Youngmin Lee, Hyewon Jun, Seoyeon Park, Deuk Young Kim, Sejoon Lee

**Affiliations:** 1 Department of Semiconductor Science, Dongguk University-Seoul, Seoul 04620, Republic of Korea; ymlee@dongguk.edu (Y.L.); dykim@dongguk.edu (D.Y.K.); 2 Quantum-Functional Semiconductor Research Center, Dongguk University-Seoul, Seoul 04620, Republic of Korea

**Keywords:** quantum dot, Coulomb blockade, single-electron tunneling, transport mechanism

## Abstract

The transport characteristics of a gate-all-around Si multiple-quantum-dot (QD) transistor were studied by means of experimental parametrization using theoretical models. The device was fabricated by using the *e*-beam lithographically patterned Si nanowire channel, in which the ultrasmall QDs were self-created along the Si nanowire due to its volumetric undulation. Owing to the large quantum-level spacings of the self-formed ultrasmall QDs, the device clearly exhibited both Coulomb blockade oscillation (CBO) and negative differential conductance (NDC) characteristics at room temperature. Furthermore, it was also observed that both CBO and NDC could evolve along the extended blockade region within wide gate and drain bias voltage ranges. By analyzing the experimental device parameters using the simple theoretical single-hole-tunneling models, the fabricated QD transistor was confirmed as comprising the double-dot system. Consequently, based on the analytical energy-band diagram, we found that the formation of ultrasmall QDs with imbalanced energetic natures (i.e., imbalanced quantum energy states and their imbalanced capacitive-coupling strengths between the two dots) could lead to effective CBO/NDC evolution in wide bias voltage ranges.

## 1. Introduction

Recently, to manipulate the vast amount of electronic information data (e.g., big data processes, artificial intelligence, the Internet of Things, etc.), many research groups have devoted their work to demonstrating how novel semiconductor quantum-dot (QD) devices can realize future quantum computations [1,2,3,4,5]. Among various semiconductor QD devices, the silicon (Si) QD-based single-electron (or single-hole) tunnel-junction transistor is one of the most promising device schemes because of its unique transfer and output characteristics. Namely, owing to the strong sub-band modulation in Si QDs, one could effectively demonstrate the unique features of both Coulomb blockade oscillation (CBO) and negative differential conductance (NDC) in a single device system even at room temperature [6,7,8,9,10,11]. Additionally, from both scientific and technical perspectives, the Si process platform is still powerful for driving significant advancements in future nanodevice fabrication technology. By utilizing CBO and NDC characteristics, several types of extraordinary data-processing circuits have been conceived and demonstrated on various Si QD device architectures (e.g., multi-functional logic circuits [12], multi-valued logic circuits [8,13], stochastic data-processing circuits [14,15], quantum cellular automata [16], etc.). To establish a tangible application platform of such astonishing functionalities, the precise control of single-electron (or single-hole) tunneling must be satisfied, particularly at an elevated temperature above 300 K. In other words, electrical one-electron (or one-hole) addition energy (i.e., charging/discharging energy for single-electron (or one-hole) tunneling into/from the Si QD) should be precisely controlled by changing the external bias voltages of the fabricated QD tunnel-junction transistor. Typically, the dot potential of the three-terminal QD transistor is controlled by the gate voltage (V_G_) and the drain voltage (V_D_) of the QD transistor so that the CBO and NDC behaviors can take place via one-by-one single-electron (or one-hole) tunneling through the quantum energy states. In lithographically patterned CMOS-compatible Si QD transistors, however, co-tunneling effects and thermally activated carrier conduction often occur because of the parasitic metal-oxide-semiconductor field-effect transistor (MOSFET) [17,18,19]. To solve this issue, in our previous studies we investigated effective ways to reduce thermal quenching and thermal broadening of CBO and NDC by forming an ellipsoidal Si QD structure [8,20] and/or constructing a Si multi-QD system [12,21]. The former utilizes the large quantum-level spacings of the ellipsoidal Si single-QD system, and the latter uses the reduced electron temperature in the multiple-tunnel-junction system. To take full advantage of these Si QD transistors, as a primary task, one needs to understand their transport mechanisms. In the case of single-QD systems, the single-electron (or single-hole) transport mechanisms are well understood due to many substantial studies being conducted on both theoretical models and experimental analyses [22,23,24,25,26,27,28]. However, in the case of the multi-QD devices, experimental understandings of their transport behaviors were somewhat restricted because of stochastic tunneling events. Therefore, it is essential to find an easy approach that can enable experimental understandings of the single-electron (or single-hole) transport mechanisms in the multi-QD transistors.

Based upon all of the above background information, we fabricated a Si multi-QD transistor and characterized its transport mechanisms by means of experimental parametrization, which was fully based on the theoretical single-hole-tunneling model. The device was devised in the form of the gate-all-around (GAA) Si nanowire-channel MOSFET, in which self-assembled multiple Si QDs were created along the Si nanowire channel. Herein, the single-hole transport characteristics of the fabricated Si multi-QD transistor are thoroughly examined and discussed through comprehensive studies on both experimental characterization and theoretical analysis.

## 2. Experimental Section

**Figure 1:**
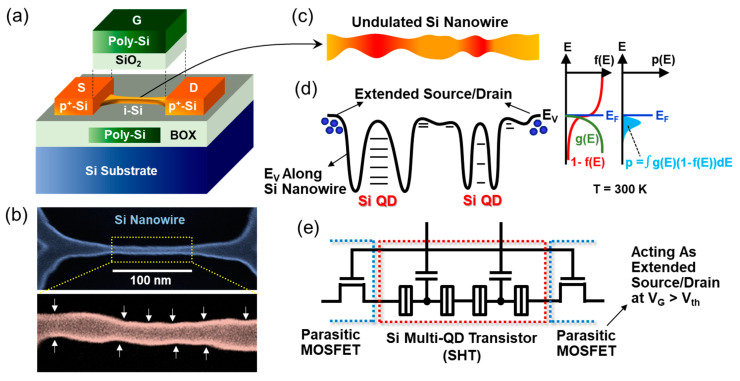
(**a**) Schematic configuration of the fabricated Si multi-QD transistor. (**b**) SEM image of the volumetrically undulated Si nanowire channel. (**c**) Schematic illustration of the undulated Si nanowire channel. (**d**) Expected energy-band diagram at the valence band region of the undulated Si nanowire channel and its corresponding Fermi–Dirac distribution function, density of state function, and carrier distribution function. E_V_ and E_F_ denote the valence band and the Fermi level, respectively. (**e**) Equivalent circuit composed of the active device of the Si multi-QD transistor with two parasitic MOSFETs at S/D regions.

Figure 1a schematically illustrates the device configuration of the fabricated Si multi-QD transistor, which was constructed in the form of the CMOS-compatible GAA Si nanowire-channel MOSFET. To devise such an architecture, firstly, the [100] Si nanowire channel (*l*: 200 nm, *w*: 40 nm) was patterned by *e*-beam lithography onto the silicon-on-insulator substrate (*t*_Si_: 10 nm). Subsequently, the volumetric size of the patterned Si nanowire was further reduced by isotropic wet etching using the SC-1 solution (NH_4_OH:H_2_O_2_:H_2_O = 1:1:6). Through this step, the width of the Si nanowire was shrunk to be approximately 10 nm (Figure 1b). Next, with the aim of constructing the GAA structure, a part of the buried oxide (i.e., SiO_2_ underneath the Si nanowire) was removed through oxide etching by using diluted hydrogen fluoride (HF:H_2_O = 1:10). After this step, only a small part of the Si nanowire channel was suspended from the buried oxide because the large source (S) and drain (D) areas could still support the beam shape of the Si nanowire channel. To create the gate oxide of the GAA MOSFET structure, the surface of the suspended Si nanowire channel was then oxidized through dry oxidation at 900 °C. Due to oxidation of the Si nanowire surface, the cross-sectional diameter of the Si nanowire (*d*_NW_) was eventually further decreased down to approximately 5 nm [6,29]. Thereafter, the formation of the GAA stack was completed by depositing the additional layers of SiO_2_ (approximately 30 nm) and polycrystalline n^+^-Si (approximately 250 nm). Finally, the S and D reservoirs (*p* ≈ 10^20^ cm^−3^) were formed by BF_2_^+^ ion implantation and thermal activation at 950 °C.

## 3. Results and Discussion

Prior to discussing the electrical characteristics of the fabricated device, we will explain our device fabrication method that was employed to perform the room-temperature operation of the fabricated Si QD transistor. We obtained the self-created Si QDs through oxidation of the volumetrically undulated ultra-narrow Si nanowire. Notably, no intentional design of the QD sites and sizes was employed in our experiment. According to previous studies [30,31,32,33,34,35], there are two main approaches to devise two types of high-performance Si QD transistors: one is Si QD transistors based on epitaxially grown Si QDs [30,31,32], and the other is lithographically fabricated Si QD transistors [30,31,32]. Although the former could allow for the operation of the fabricated devices at elevated temperatures, the method is inappropriate for practical applications because of difficulties in both size and site controls. On the other hand, the latter is useful for controlling both the size and the site of the QDs. Hence, many researchers have devoted their studies to fabricating high-performance Si QD transistors by using lithography techniques. For example, forming the double/multiple barrier tunnel junctions by pattern-dependent oxidation (PADOX) [36,37,38] and creating the electrical dots/barriers by side-gate formation [39,40,41] are typical examples that can enable the fabrication of high-performance CMOS-compatible Si QD transistors. When considering the device operation temperature, however, these techniques do not satisfy the conditions for room-temperature operation because conventional lithography techniques only allow for the formation of large QD sizes and small tunnel barrier heights. We therefore adopted the QD self-formation method by using a volumetrically undulated ultra-narrow Si nanowire. Although the proposed method cannot ensure the precise size/site/number control of the Si QDs, we believe the method will be good for use in future CMOS-compatible Si QD device technology. This is because the method used here is already compatible with CMOS fabrication processes and can be further developed when advanced nanolithography techniques are well established in the near future. In other words, if the intentional patterning of the regularly undulated sub-5 nm Si nanowire is available when using the advanced lithography techniques (e.g., extreme ultraviolet lithography [42,43], heavy-ion lithography [44], scanning probe lithography [45], block copolymer self-assembly [46], etc.), one can easily control the number, sizes, and sites of the Si QDs by employing the PADOX process method as well.

**Figure 2:**
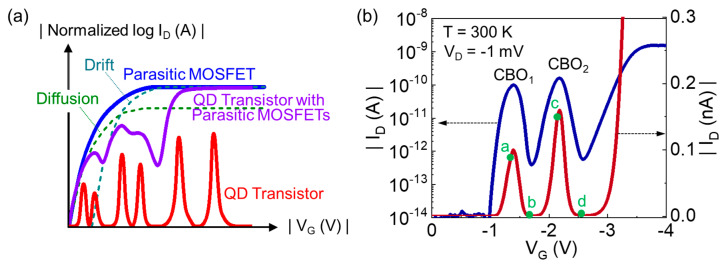
(**a**) Normalized I_D_ for the Si multi-QD transistor, parasitic MOSFET, and fabricated device including both the Si multi-QD transistor and the parasitic MOSFET. (**b**) Transfer characteristic curves of the fabricated device (i.e., I_D_–V_G_ at V_D_ = −1 mV).

Next, we interpreted the QD nature inside the volumetrically undulated ultra-narrow Si nanowire channel. As schematically illustrated in Figure 1c, during the isotropic wet-etching process, the magnitude of *d*_NW_ fluctuated along the undulated Si nanowire direction. Here, it can be noticed that such a volumetric *d*_NW_ fluctuation is much more significant after thermal oxidation of the Si nanowire surface. Thus, the *d*_NW_ sizes at the squeezed regions are much smaller than 5 nm because the *d*_NW_ at the central Si nanowire area was confirmed to be approximately 5 nm [6,29]. According to the quantum-mechanical energy-band calculation [47,48,49], the sub-band modulation in the [100] Si nanowire significantly increases with decreasing *d*_NW_. For instance, when *d*_NW_ is <2 nm in the [100] Si nanowire, the ground state is located at more than 400 meV below the valence band (E_V_) of the bulk Si [8,48]. In the present type of the volumetrically undulated Si nanowire channel, therefore, the Si multi-QD system can be self-assembled along the nanowire. In other words, the large potential barriers are formed at the squeezed Si nanowire areas (i.e., *d*_NW_ << 5 nm), and the quantum dots are created at the rest areas (i.e., *d*_NW_ ≈ 5 nm), as represented in Figure 1d. This allows the fabricated GAA Si nanowire-channel MOSFET to act as the Si multi-QD transistor. At this point, one can disregard the presence of large dots since their quantum-level spacings are too small to significantly contribute to the tunneling transport characteristics. In short, at 300 K, both the large thermal fluctuation at the bigger dots and the broad hole carrier distribution at the S/D reservoirs smear the presence of the large dots. Additionally, it should be remembered that the GAA gate stacks cover the nanowire edges at the S/D and multi-QD regions. Thus, these two nanowire edges (i.e., extended S/D regions) play a role as the parasitic MOSFET (Figure 1e), which may eventually give rise to the increase in the valley current of the present CMOS-compatible GAA MOSFET-based Si multi-QD transistor. For example, when the gate bias voltage (V_G_) is greater than the threshold voltage (V_th_) of the parasitic MOSFET, the drift current component of the parasitic MOSFET largely contributes to the increase in total drain current (I_D_) of the actual device (Figure 2a). This would, in turn, smear out the CBO features at the higher V_G_ region.

Despite such a parasitic MOSFET effect, the fabricated Si multi-QD transistor clearly exhibits two large CBO peaks at 300 K in its I_D_–V_G_ characteristic curves (Figure 2b). The magnitudes of the peak-to-valley current ratio (PVCR) are 261 and 288 for the first CBO_1_ and the second CBO_2_, respectively. In addition, the values of the full width at half-maximum (FWHM) are approximately 192 and 188 mV for CBO_1_ and CBO_2_, respectively. In principle, for the room-temperature observation of such clear CBO characteristics, the one-electron (or hole)-addition energy (*E_a_*) should be sufficiently larger than the thermal energy at room temperature (i.e., *E_th_* ≈ 26 meV at 300 K) [20,50]. *E_a_* is given by

(1) $$E_{a}=E_{C}+\Delta \varepsilon$$

(2) $$E_{C}=q^{2}/C_{QD}$$

(3) $$C_{QD}=2\pi \varepsilon \varepsilon_{0}d_{QD}$$

where *E_C_*, Δ*ε*, *q*, *C_QD_*, *ε*, *ε*_0_, and *d_QD_* are the charging energy, quantum-level spacing, unit charge, QD capacitance, dielectric constant of SiO_2_, and vacuum permittivity, respectively. Thus, one can conclude that the present device includes ultrasmall QDs (i.e., *d_QD_* < 5 nm) inside the Si nanowire channel. Furthermore, since both PVCR and FWHM are closely related to the thermal fluctuation of the QDs [51,52], the observed large PVCR and small FWHM values obviously indicate that the present device contains ultrasmall QDs that may possess large Δ*ε* values (>>26 meV).

**Figure 3:**
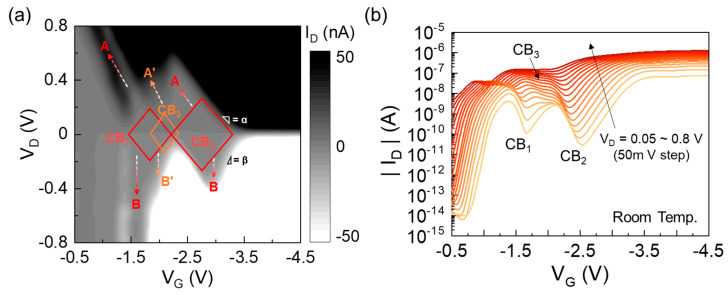
(**a**) Contour plot of I_D_ as functions of V_G_ and V_D_. (**b**) CBO evolution at the positive V_D_ region (=0.05–0.8 V).

The existence of the ultrasmall QDs can be further elucidated from the charge stability diagram of the fabricated Si multi-QD transistor. As denoted by CB_1_ and CB_2_ in Figure 3a, the device exhibits the two Coulomb blockade regions, which correspond to CBO_1_ and CBO_2_ in Figure 2b, respectively. Here, it should be noticed that both the CB_1_ and CB_2_ regions are extended toward the A and B directions. In the case of CB_1_, particularly, the extended CB regions do not disappear even at a high V_D_ up to ±0.8 V. According to our previous studies [51,53], such a long and clear CB extension is attributed to both the large quantum-level spacings and the large tunneling barrier heights. Additionally, as indicated by CB_3_, the present device shows the overlapped CB region, which is also extended toward the A’ and B’ directions. This depicts how present device comprises the multi-QD system with irregular dot-and-barrier shapes. As previously mentioned, in the present device, the multi-QD system was self-formed along the undulated Si nanowire at the volumetrically shrunken areas (i.e., squeezed regions = tunnel barriers and their adjacent areas = dots). Thus, both the quantum-level spacings and the tunnel barrier heights are inhomogeneous. This might result in imbalanced energetic CB conditions for each QD; hence, the stochastic tunneling events would occur throughout the entire multi-QD system [54]. Therefore, some parts of the adjacent CB regions could be overlapped at certain bias voltages.

The split of the CBO peaks can also verify the formation of the multi-QD system. For example, when applying a high V_D_ that can break a certain stochastic tunneling condition, the multi-QD system begins to renormalize its energetic condition for stochastic tunneling. Namely, some of the quantum states start and/or stop contributing their energetic pathways to the renormalized stochastic tunneling transport condition. Then, the CBO peaks eventually split at the high V_D_ [12]. In the present device, as can be seen from Figure 3b, the CBO peaks are split at the high V_D_ region so that the additional CB_3_ region starts appearing in between the CB_1_ and CB_2_ regions. This corresponds to the extension of the CB_3_ region toward the A’ direction, as observed in the charge stability diagram (Figure 3a). Based upon these results, one can surmise that the present device is composed of multiple Si QDs. According to both theoretical and experimental studies [21,55], the formation of the multi-QD system is helpful for reducing the co-tunneling effect so that the valley current (I_valley_) of the CBO can be decreased at the higher V_D_ region. Correspondingly, the present device exhibits the clear valley states even at a high V_D_ above 0.5 V (Figure 3b).

**Figure 4:**
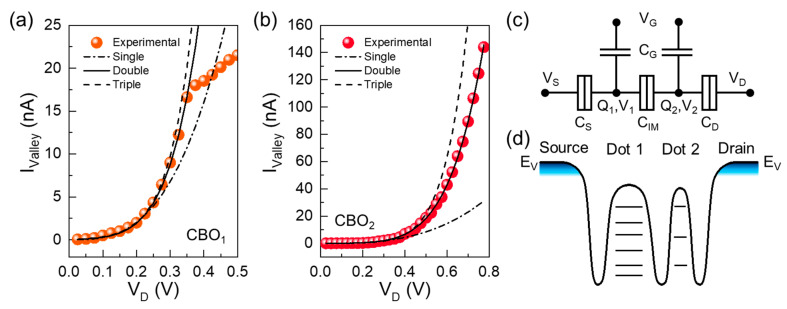
I_valley_ as a function of V_D_ for (**a**) CBO_1_ and (**b**) CBO_2_. (**c**) Equivalent circuit of the Si multi-QD transistor. (**d**) Energy-band diagram at the valence band region along the S-channel-D direction expected from fitting the experimental data to the theoretical models.

According to the co-tunneling model [21,56], I_valley_ is given by

(4) $$I_{\mathrm{valley}}\equiv G_{b}^{N+1}{\left\{{\left(eV_{D}\right)}^{2}+{\left(2\pi k_{B}T_{eff}\right)}^{2}\right\}}^{N}V_{D}$$

where $G_{b}^{N+1}$ is the conductance multiplication for every tunnel barrier, *N* is the number of QDs, *k_B_* is the Boltzmann constant, and *T_eff_* is the effective electron temperature. Therefore, one can easily deduce the number of QDs by using the above equation. For example, when *N* = 1 (i.e., single-QD system), 2 (i.e., double-QD system), and 3 (i.e., triple-QD system), I_valley_ can be described as follows:

(5) $$I_{\mathrm{valley}\left(N=1\right)}=\alpha G_{S}G_{D}\left\{e^{2}V_{D}^{3}+{\left(2\pi k_{B}T_{eff}\right)}^{2}V_{D}\right\}$$

(6) $$I_{\mathrm{valley}\left(N=2\right)}=\beta G_{S}G_{i}G_{D}\left\{e^{4}V_{D}^{5}+2e^{2}{\left(2\pi k_{B}T_{eff}\right)}^{2}V_{D}^{3}+{\left(2\pi k_{B}T_{eff}\right)}^{4}V_{D}\right\}$$

(7) $$I_{\mathrm{valley}\left(N=3\right)}=\gamma G_{S}G_{i1}G_{i2}G_{D}\left\{e^{6}V_{D}^{7}+3e^{4}{\left(2\pi k_{B}T_{eff}\right)}^{2}V_{D}^{5}+3e^{2}{\left(2\pi k_{B}T_{eff}\right)}^{4}V_{D}^{3}+{\left(2\pi k_{B}T_{eff}\right)}^{6}V_{D}\right\}$$

where *α*, *β*, and *γ* are the proportional factors, and *G_S_*, *G_i_*, and *G_D_* are the source, intermediate, and drain conductance values, respectively. By fitting the experimental I_valley_ values to Equations (5)–(7), one can find out the number of QDs in the multi-QD transistor. Figure 4a,b shows the I_valley_ values as a function of V_D_ for CBO_1_ and CBO_2_, respectively. The closed circles are the experimental I_valley_ data at each CB state, and the dot-dashed, solid, and dashed lines are the best-fitted curves obtained by using Equations (5)–(7), respectively. As can be confirmed from Figure 4a,b, the experimental I_valley_ values of CBO_1_ and CBO_2_ were well fitted only when *N* = 2. One can therefore conjecture that the present multi-QD transistor was constructed with the double-dot system.

At this point, it should be noted that the sizes and the shapes of both the dots and the barriers are inhomogeneous in the present multi-QD transistor. This strongly affects the energy-band profile of the multi-QD system. To qualitatively infer the energy-band diagram of the present device, we analyzed the capacitance ratios because these values provide electrostatic information on the dots and barriers. Firstly, we assume that, based upon the above results, the present device includes two QDs (i.e., *N* = 2). As represented in the equivalent circuit (Figure 4c), each QD is separated by tunnel barriers (i.e., *C*_S_: source-side tunnel barrier; *C*_IM_: QD-to-QD intermediate tunnel barrier; and *C*_D_: drain-side tunnel barrier) and is capacitively coupled to the GAA stack (i.e., *C*_G_: gate oxide). In this double-QD system, the total charge in each dot (*Q*_1_ or *Q*_2_) equals the sum of charges stored in all the capacitors connected to the dot, and it can be described by [19,57]

(8) $$Q_{1}=C_{S}\left(V_{1}-V_{S}\right)+C_{G}\left(V_{1}-V_{G}\right)+C_{\mathrm{IM}}\left(V_{1}-V_{2}\right)$$

(9) $$Q_{2}=C_{D}\left(V_{2}-V_{D}\right)+C_{G}\left(V_{2}-V_{G}\right)+C_{\mathrm{IM}}\left(V_{2}-V_{1}\right)$$

where V_1_ and V_2_ are the electrostatic potentials for QD_1_ and QD_2_, respectively. Accordingly, V_1_ and V_2_ can also be determined by the following relationship:

(10) $$\left(\begin{array}{c} V_{1}\\ V_{2} \end{array}\right)=\frac{1}{C_{1}C_{2}-C_{\mathrm{IM}}^{2}}\left(\begin{array}{cc} C_{2} & C_{\mathrm{IM}}\\ C_{\mathrm{IM}} & C_{1} \end{array}\right)\times \left(\begin{array}{c} Q_{1}+C_{S}V_{S}+C_{G}V_{G}\\ Q_{2}+C_{D}V_{D}+C_{G}V_{G} \end{array}\right)$$

where *C*_1_ and *C*_2_ are the total electrostatic capacitances of QD_1_ and QD_2_ at the charged states under the given bias voltages (i.e., *C*_1_ = *C*_G_ + *C*_S_ + *C*_IM_ and *C*_2_ = *C*_G_ + *C*_D_ + *C*_IM_), respectively. Here, it should be noted that the CB state begins to appear when the electrostatic potentials of the two electrical dots are the same. When assuming V_1_ = V_2_, Equation (10) can be solved as follows:

(11) $$\left(C_{2}-C_{1}\right)C_{G}V_{G}=\left(C_{1}-C_{\mathrm{IM}}\right)C_{D}V_{D}-\left(C_{2}-C_{\mathrm{IM}}\right)C_{S}V_{S}$$

From Equation (11), therefore, the electrostatic charge states at given bias conditions can be described by the following relationships:

(12) $$\frac{V_{S}}{V_{G}}=-\frac{\left(C_{2}-C_{1}\right)C_{G}}{\left(C_{2}-C_{\mathrm{IM}}\right)C_{S}}=-\alpha \;\left(\mathrm{for}\;D\;\mathrm{grounded}\;\mathrm{state};i.e.,{\mathrm{when}\;V}_{D}=0\right)$$

(13) $$\frac{V_{D}}{V_{G}}=\frac{\left(C_{2}-C_{1}\right)C_{G}}{\left(C_{1}-C_{\mathrm{IM}}\right)C_{D}}=\beta \;\left(\mathrm{for}\;S\;\mathrm{grounded}\;\mathrm{state};i.e.,{\;\mathrm{when}\;V}_{S}=0\right)$$

Based on the above model, we determined the magnitudes of |*α*| (=0.73) and |*β*| (=1.6) from the charge stability diagram (Figure 2a). When considering that the total capacitances of the electrical dots are *C*_1_ = *C*_G_ + *C*_S_ + *C*_IM_ and *C*_2_ = *C*_G_ + *C*_D_ + *C*_IM_, the obtained result of |*α*/*β*| < 0 represents the fact that *C*_S_ is greater than *C*_D_ (i.e., |*α*/*β*|∝*C*_D_/*C*_S_ < 0). Again, since *C*_S_ > *C*_D_, it can be concluded that *C*_1_ > *C*_2_ because both *C*_G_ and *C*_IM_ are common in the double-QD system. By means of the above analysis, consequently, one can expect that the source-side QD is larger than the drain-side QD in our Si double-QD transistor. Considering that the smaller QD possesses the lager Δ*ε*, the expected potential profile of the present device can be drawn with a bigger source-side QD (i.e., smaller Δ*ε*) and a smaller drain-side QD (i.e., larger Δ*ε*) (see the valence band profile in Figure 4d).

**Figure 5:**
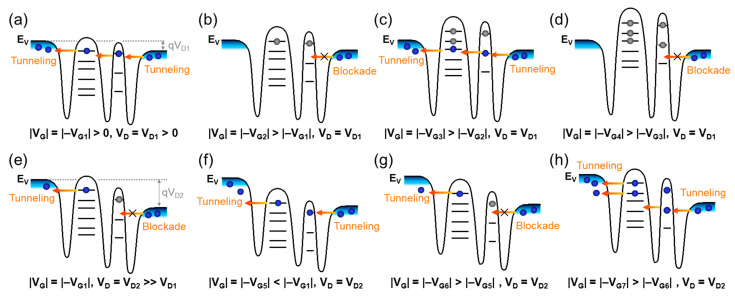
Carrier transport mechanisms of CBO for the double-QD transistor represented in the energy-band diagrams at various bias conditions: (**a**) |V_G_| = |−V_G1_| > 0 and V_D_ = V_D1_ > 0, (**b**) |V_G_| = |−V_G2_| > |−V_G1_| and V_D_ = V_D1_, (**c**) |V_G_| = |−V_G3_| > |−V_G2_| and V_D_ = V_D1_, (**d**) |V_G_| = |−V_G4_| > |−V_G3_| and V_D_ = V_D1_, (**e**) |V_G_| = |−V_G1_| and V_D_ = V_D2_ >> V_D1_, (**f**) |V_G_| = |−V_G5_| < |−V_G1_| and V_D_ = V_D2_, (**g**) |V_G_| = |−V_G6_| > |−V_G5_| and V_D_ = V_D2_, and (**h**) |V_G_| = |−V_G7_| > |−V_G6_| and V_D_ = V_D2_.

By using the above analytical energy-band diagram (Figure 4d), we can interpret the possible carrier transport mechanisms of the fabricated Si multi-QD transistor. First, we will explain the double CBO characteristics of the present device. Prior to discussing the carrier transport mechanism, it is important to note that the dot potential is mostly governed by V_G_ rather than V_D_ because of the strong capacitive coupling from the GAA structure [29,58]. Thus, we firstly assume that the double-QD transistor is set at specific bias conditions of a moderate |−V_G1_| and low V_D1_, at which the initial stage of single-hole tunneling can take place (Figure 5a). At this stage, the hole carrier can be transferred from D to S via the quantum states of QD_2_ and QD_1_. When increasing the magnitude of −V_G2_ (e.g., |−V_G2_| > |−V_G1_|) while maintaining V_D_ at low V_D1_, the dot potential decreases. Then, the Si double-QD transistor would be set on the blockade state due to the large Δ*ε* in QD_2_ (Figure 5b). As one keeps increasing the magnitude of −V_G_ (e.g., |−V_G3_| > |−V_G2_|) at low V_D1_, the device needs to be set in the on-state to allow single-hole tunneling from D to S via QD_2_ and QD_1_ (Figure 5c). When further increasing V_G_ from |−V_G3_| to |−V_G4_|, the blockade state would appear again because of the large Δ*ε* in QD_2_ (Figure 5d). This may allow us to observe the double CBO features from the fabricated device, as depicted in Figure 2b. In short, Figure 5a–d corresponds to the operation points a–d depicted in Figure 2b, respectively.

Next, we explain the V_D_-dependent CBO evolution, which can be observed in Figure 3a,b. For this, firstly, we assume that the device was set on the initial single-hole-tunneling stage at |−V_G1_| and V_D1_ (i.e., Figure 5a). If one increases V_D_ from V_D1_ to V_D2_, the dot potential also increases (Figure 5e) because the capacitive coupling between QD and D would be no longer negligible at a high V_D_ [17]. At this stage, the single-hole-tunneling event would suddenly stop because the imbalanced capacitive-coupling strengths between G-QD and QD-D would break the tunneling condition (i.e., blockade state). To perform the single-hole-tunneling transport under the same V_D2_ condition, the dot potential should be increased until the D-side Fermi level can meet the first excited quantum state of QD_2_. Therefore, one needs to decrease the magnitude of −V_G_ from |−V_G1_| to |−V_G5_| (where 0 < |−V_G5_| < |−V_G1_|) when V_D_ is increased from V_D1_ to V_D2_ (Figure 5f). Namely, when V_D_ is positively increased, the V_G_ bias point should also be positively shifted to satisfy the tunneling-on condition via the first excited state. This eventually leads to the CBO shift toward the A and A’ directions, as already observed in Figure 3a,b. From this bias point (i.e., |−V_G5_| and V_D2_), one can demonstrate the multiple CBO peaks by increasing the magnitude of |−V_G_| over |−V_G5_|. For example, at the same V_D2_, the second and/or the third CBO peaks can be demonstrated by repeating the tunneling-off (i.e., |−V_G6_| > |−V_G5_|, Figure 5g) and tunneling-on (i.e., |−V_G6_| > |−V_G5_|, Figure 5h) conditions. Here, it should be also noted that the overall quantum energy states are energetically split between QD_1_ and QD_2_ because of the high V_D2_. Namely, the overall dot potential of QD_1_ is lower than that of QD_2_ due to the high V_D_. This eventually gives rise to the increase in co-tunneling events because of the energetic imbalance between QD_1_ and QD_2_. As a result, the device exhibits the extra CBO peak through the renormalization of stochastic tunneling conditions at a higher V_D_ (e.g., CB_3_ in Figure 3a,b).

**Figure 6:**
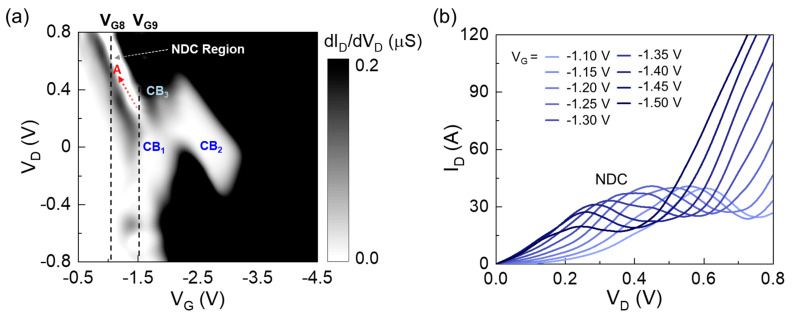
(**a**) Contour plots of dI_D_/dV_D_ as functions of V_G_ and V_D_. (**b**) I_D_–V_D_ characteristic curves at V_G_ of −1.1–−1.5 V.

The existence of an ultrasmall QD (i.e., large Δ*ε*) may allow us to observe the clear NDC features in the output characteristics of the QD transistor. To trace the NDC behaviors, we examined the differential drain conductance (dI_D_/dV_D_) characteristics of the present device. Figure 6a shows the contour plot of dI_D_/dV_D_ as functions of V_G_ and V_D_. The device clearly reveals the NDC region. For example, with the increasing V_D_ at a certain V_G_ (e.g., V_G8_ and V_G9_), the color of dI_D_/dV_D_ is changed into “white-gray-white-black”, which is indicative of the sudden drop in drain conductance at a certain V_D_ point (i.e., NDC). Interestingly, the NDC region is also extended toward the A direction (Figure 6a). Correspondingly, the NDC peaks shift toward the low V_D_ region as |−V_G_| increases (Figure 6b). These features are analogous to those of CBO, as observed through the CBO evolution along the extended CB region (Figure 3a,b).

**Figure 7:**
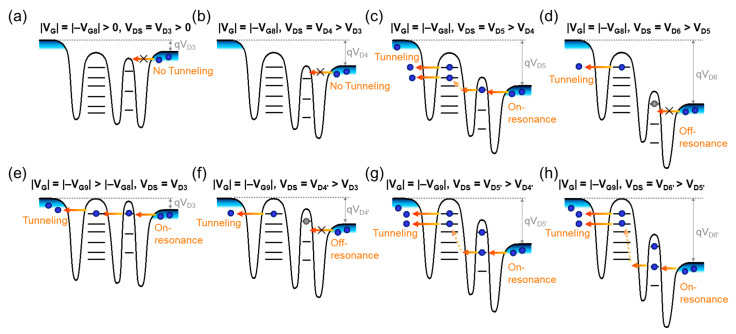
Carrier transport mechanisms of NDC for the double-QD transistor represented in the energy-band diagrams at various bias conditions: (**a**) |V_G_| = |−V_G8_| > 0 and V_DS_ = V_D3_ > 0, (**b**) |V_G_| = −|V_G8_| and V_DS_ = V_D4_ > V_D3_, (**c**) |V_G_| = |−V_G8_| and V_DS_ = V_D5_ > V_D4_, (**d**) |V_G_| = |−V_G8_| and V_DS_ = V_D6_ > V_D5_, (**e**) |V_G_| = |−V_G9_| > |−V_G8_| and V_DS_ = V_D3_, (**f**) |V_G_| = |−V_G9_| and V_DS_ = V_D4′_ > V_D3_, (**g**) |V_G_| = |−V_G9_| and V_DS_ = V_D5′_ > V_D4′_, and (**h**) |V_G_| = |−V_G9_| and V_DS_ = V_D6′_ > V_D5′_.

The NDC evolution can also be explained by using the aforementioned analytical energy-band diagram. Firstly, we interpret the NDC mechanism of the present device at a low V_G_ (e.g., V_G_ = |−V_G8_| > 0). At this V_G_ bias condition, no hole carriers could transfer from D into QD_2_ because the Fermi level of D is far from the first excited state of QD_2_ (Figure 7a). In other words, due to the large Δ*ε* of QD_2_, it is hard to perform the resonance state when both V_D_ and V_G_ are low. The similar situation is maintained unless the magnitude of V_D_ is increased to match the on-resonance condition (Figure 7b,c). When increasing V_D_ after the on-resonance at the first excited state, the tunneling event is prohibited again because of the large Δ*ε* between the first and the second excited quantum states (Figure 7d). Thus, one can surmise that the NDC could occur at the relatively high V_D_ region when V_G_ is low. Next, we explain the NDC evolution toward the lower V_D_ region, which was observed when the higher V_G_ was applied (Figure 6b). Here, let us assume that the device was set at the on-resonance state at an increased V_G_ (i.e., |−V_G9_| > |−V_G8_|), even if the magnitude of V_D_ is the same as the initial V_D3_ above (Figure 7e). From this bias point, if one increases the magnitude of V_D_ (i.e., V_D_ = V_D4′_ > V_D3_), the resonance state is immediately changed from ‘on’ to ‘off’ (Figure 7f). When increasing V_D_ more from V_D4′_ to V_D5′_, the second on-resonance state occurs at the second excited state (Figure 7g). At a high V_D_ (e.g., V_D6′_ > V_D5′_), the on-resonance state would be retained because, at the high V_D_, the dot potential would also be capacitively coupled to the drain potential (Figure 7g). As a result, the NDC can occur at the relatively lower V_D_ region when a higher V_G_ is applied to the device. To briefly summarize, the NDC evolution would take place toward the low V_D_ region as V_G_ increases.

## 4. Summary and Conclusions

We fabricated a high-performance room-temperature-operating Si multi-QD transistor in the form of the CMOS-compatible GAA Si nanowire-channel MOSFET. Due to the formation of ultrasmall QDs (i.e., large Δ*ε*) inside the volumetrically undulated Si nanowire channel, the device clearly exhibited multiple CBO features at 300 K. Owing to the formation of GAA (i.e., strong capacitive coupling to the gate), the device displayed CBO evolution at wide V_D_ and V_G_ ranges toward the extended CB region. Because of the large Δ*ε* in the self-formed Si QDs, furthermore, the device not only clearly revealed the NDC oscillation peak but also showed its evolution within wide V_D_ and V_G_ ranges. Through experimental parametrization by using the theoretical models, it was found that the fabricated device involves two predominantly ultrasmall QDs. Based upon the analytical energy-band diagram, the carrier transport mechanisms were comprehensively interpreted. Consequently, the present study provides a simple analysis method (i.e., analysis of experimental device parameters in terms of simple theoretical models), which can allow an easy understanding of the experimental single-charge transport behaviors in the multi-QD transistors, holding great promise for future nanoelectronic information technology.

## Data Availability

Not applicable.
